# Effect of Treatment with Ginger on the Severity of Premenstrual Syndrome Symptoms

**DOI:** 10.1155/2014/792708

**Published:** 2014-05-04

**Authors:** Samira Khayat, Masoomeh Kheirkhah, Zahra Behboodi Moghadam, Hamed Fanaei, Amir Kasaeian, Mani Javadimehr

**Affiliations:** ^1^Pregnancy Health Research Center, Zahedan University of Medical Sciences, Zahedan, Iran; ^2^Department of Reproductive Health, School of Nursing and Midwifery, Tehran University of Medical Sciences, Tehran, Iran; ^3^Department of Reproductive Health, School of Nursing and Midwifery, Shahid Beheshti University of Medical Sciences, Tehran, Iran; ^4^Department of Medical Education, Iran University of Medical Sciences, Tehran 1419733171, Iran; ^5^School of Nursing and Midwifery, Iran University of Medical Sciences, Tehran 1419733171, Iran; ^6^Department of Physiology, School of Medicine, Zahedan University of Medical Sciences, Zahedan, Iran; ^7^Non-Communicable Diseases Research Center, Endocrinology and Metabolism Population Sciences Institute, Tehran University of Medical Sciences, Tehran, Iran; ^8^Department of Epidemiology and Biostatistics, School of Public Health, Tehran University of Medical Sciences, Tehran, Iran; ^9^Department of Medical English Language, School of Medicine, Zahedan University of Medical Sciences, Zahedan, Iran

## Abstract

Premenstrual syndrome (PMS) is a common disorder. Although the etiology of PMS is not clear, to relieve from this syndrome different methods are recommended. One of them is use of medicinal herbs. This study was carried out to evaluate effects of ginger on severity of symptoms of PMS. This study was a clinical trial, double-blinded work, and participants were randomly allocated to intervention (*n* = 35) and control (*n* = 35) groups. To determine persons suffering from PMS, participants completed daily record scale questionnaire for two consecutive cycles. After identification, each participant received two ginger capsules daily from seven days before menstruation to three days after menstruation for three cycles and they recorded severity of the symptoms by daily record scale questionnaire. Data before intervention were compared with date 1, 2, and 3 months after intervention. Before intervention, there were no significant differences between the mean scores of PMS symptoms in the two groups, but after 1, 2, and 3 months of treatment, there was a significant difference between the two groups (*P* < 0.0001). Based on the results of this study, maybe ginger is effective in the reduction of severity of mood and physical and behavioral symptoms of PMS and we suggest ginger as treatment for PMS.

## 1. Introduction

Premenstrual syndrome (PMS) is one of the most common problems in women at their reproductive age [1, 2]. PMS is defined as the recurrent mood and physical symptoms which is usually in the luteal phase, and it remits in the follicular phase of the menstrual cycle [3–6]. There is a high prevalence of PMS; about 80% of women reported mild premenstrual symptoms, 20%–50% reported moderate symptoms, and about 5% of women had severe symptoms [7, 8]. Despite the high incidence of premenstrual syndrome, causes of it have not been clear and several etiologies have been suggested (e.g., hormonal change, neurotransmitters, prostaglandins, diet, drugs, and lifestyle) [7, 9–14]. Although the exact etiology of PMS is not known, a wide range of therapeutic interventions have been tested to treat premenstrual symptoms (e.g., lifestyle changes, pharmacological intervention, and nonpharmacological treatments) [15–22]. Due to the side effects of chemical drugs, except severe cases, chemical drugs consumption is not recommended. Today, complementary and herbal medicine are commonly used in the treatment of many chronic conditions such as PMS, menopausal symptoms, and dysmenorrhea [17, 20, 23–27].

Ginger is commonly used in the herbal medicine and traditionally it used to treat dysmenorrhea [28, 29].* Zingiber officinale* is the scientific name of plant Ginger [30]. Ginger rhizome is used in traditional medicine [31]. Studies showed beneficial effects of ginger on vomiting, nausea, motion sickness, arthritis, migraine, headache, and so forth [32]. In addition, studies have shown that the ginger can modulate prostaglandins system [33–35]. With regard to the role of prostaglandins in PMS and effect of ginger on modulation of prostaglandins system, the aim of this study was evaluating the effects of ginger on the severity of the PMS symptoms.

## 2. Materials and Methods

This clinical trial was a randomized, double-blinded, and placebo-controlled study. The study was done on 70 female students in dormitories of Tehran University of Medical Sciences in the year of 2013. Data were collected during 7 months. The ethics committee of Tehran University of Medical Sciences approved the study (No. 97/130/D/92) and it was registered at Iranian Registry of Clinical Trial (IRCT No. 201301012751N7).

Inclusion criteria were as follows: being healthy premenopausal women aged between 18 and 35 years, having regular menstrual cycles of 21–35 days, being single, lacking sensitivity to ginger, not taking any medication, not drinking alcohol, not smoking, and not having stressful events in the last 3 months.

The participants recorded symptoms with daily record questionnaires (this form contains a table with 19 symptoms of premenstrual syndrome questionnaire based on the DSM-IV (the fourth edition of the Diagnostic and Statistical Manual of Mental Disorders of the American Psychiatric Association), Self-Rating Scale) for two cycles before the intervention. This questionnaire determines the severity of PMS using 3 items, including mood symptoms (restlessness; irritability; anxiety, depression, or sadness; crying; feeling of isolation), physical symptoms (headache, breast tenderness, backache, abdominal pain, weight gain, swelling of extremities, muscle stiffness, gastrointestinal symptoms, and nausea), and behavioral characteristics (fatigue, lack of energy, insomnia, difficulty in concentrating, increased or decreased appetite). Then the severity of premenstrual syndrome was evaluated for all participants ((0) absence of symptoms, (1) mild symptoms that may not interfere with everyday activities, (2) moderate symptoms that interfere with daily activities, and (3) severe symptoms that impede doing daily activities). Each participant with at least five symptoms was finally diagnosed as a person with PMS. After identifying patients with PMS, they were randomly divided into two groups (*n* = 35). The first group received ginger capsule and the second group received placebo capsule. Doses of ginger and placebo were 250 mg/12 hours and given from 7 days before and until 3 days after onset of menstrual bleeding. The subjects completed the daily record questionnaire at their first, second, and third menstrual cycles. After interventions, again the severity of PMS was evaluated. Participants and researcher were blind about the kind of drug throughout the study. Exclusion criteria were incidence of side effects drugs or drugs allergy, use of other drugs, drug use disorder, identifying any diseases during the study, getting married during the study, menstrual irregularities, and irregular bleeding event during the study.

Statistical analysis was performed using software SPSS-18. Intergroup analyses were performed to compare the total score of PMS, mood, and behavioral and physical symptoms before and after the intervention. Intergroup analysis was performed by independent samples *t*-test. *P* < 0.05 was considered significant.

## 3. Results

**Figure 1:**
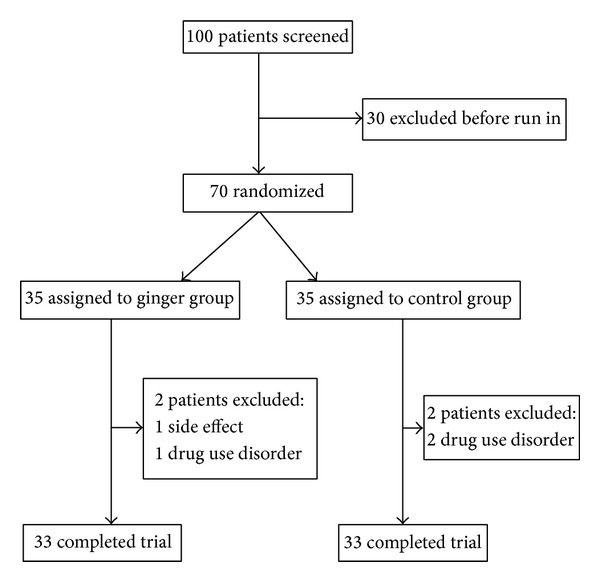
Trial profile.

Out of the 70 students who participated in the study 2 persons in the ginger group and 2 persons in the placebo group did not complete the study (Figure 1). In the ginger group two samples were excluded, one because of incidence of side effect and second one due to misuse of ginger. In the placebo group reason for exclusion for each of the 2 people was drug use disorder.

No significant differences between two groups were seen with regard to demographic characteristics and menstrual history data (Table 1).

There were no significant differences regarding the total score of PMS, severity of mood, and physical and behavioral symptoms between the two groups before the intervention (resp., *P* = 0.71, *P* = 0.69, *P* = 0.56, and *P* = 0.76) (Table 2). Statistical analysis demonstrated that there were significant differences in total score of PMS, severity of mood, and physical and behavioral symptoms between the two groups after the intervention, and ginger could reduce severity symptom of PMS (Table 2).

## 4. Discussion

The findings of this study showed that ginger could significantly reduce the total score of PMS, severity of mood, and physical and behavioral symptoms of the first month intervention. So according to the availability and the safety of ginger, the ginger can be an appropriate treatment in reducing symptoms of premenstrual syndrome. One of the proposed mechanisms that lead to premenstrual syndrome is change in prostaglandin system [7, 16, 36]. Ginger through the inhibition of the metabolism of cyclooxygenase and lipoxygenase prevents the production of prostaglandins [35]. To our knowledge, this study is the first report about the effect of ginger on premenstrual syndrome. Rahnama et al. [28] and Ozgoli et al. [29] showed that the use of ginger is effective in reducing symptoms of dysmenorrheal [28, 29]. Primary dysmenorrhea is caused by excessive production of prostaglandins from the endometrial tissue, and 80% of cases of dysmenorrhea with prostaglandin inhibitors can be improved [29]. Because some ginger compounds are prostaglandin inhibitors and are effective on dysmenorrhea, so perhaps through this mechanism they could be effective on other problems of menstruation cycle [28, 29]. Some of symptoms of PMS like pain are common with dysmenorrhea (such as backache and abdominal pain). In our study these symptoms of PMS have been reduced.

In women with PMS, severe headache outbreaks during the luteal phase and an increase in headache resistance to painkillers and anti-inflammatory drugs lead to increase in depression, irritability, anxiety, anger, and food intolerance during the luteal phase [37–39]. Cady et al. [40] reported that ginger administration caused a significant deceleration in severity of headache in patients with migraine [40]. One of the symptoms of PMS is incidence of headache and exacerbation of migraine. In our study ginger was found to be effective in relieving headaches.

Levine et al. reported that the ginger reduced the delayed nausea of chemotherapy and reduced use of antiemetic medications [41]. Ginger was effective in treating nausea and vomiting during pregnancy [31, 42]. Study of K. S. Naeine and S. S. Naeine [43] showed that the use of 1 gram of powdered ginger, 1 hour before taking contraceptive pills, induced reduction in contraceptive pills nausea [43]. Nausea is one of symptoms of PMS; in our study the incidence of gastrointestinal disturbances and nausea after taking ginger reduced.

Studies showed that ginger has anti-inflammatory properties and is effective in treatment of pain in patients with osteoarthritis, muscle pain [30, 33, 44]. Joint and muscle pain are also symptoms of PMS; in the present study, ginger was effective in relieving severity of these symptoms of PMS.

The only side effect observed in the present study was nausea in the group of ginger. In addition, the use of ginger in traditional medicine to treat colds, fever, sore throat, nausea, stomach upset, muscle aches, and arthritis and for cancer prevention is effective [32]. Therefore, the use of ginger in the treatment of PMS can also benefit from other advantages of ginger.

## 5. Conclusions

The findings of the present study demonstrated the likelihood of usefulness of ginger in treating PMS while no specific side effects have been seen, and consumption of it is associated with health benefits. Ginger is readily available because of its low cost and, so, can be widely used in the treatment of premenstrual syndrome. According to increased tendency in the use of traditional medicine and herbal medicine, especially for people who have no desire to use chemical drugs, with more side effects, use of ginger, with very low side effects, is very useful. Furthermore, we cannot find study that evaluates effect of ginger on PMS; we suggest doing more studies with larger number of patients concerning the efficacy and safety of different doses of ginger.

## Figures and Tables

**Table 1:** The demographic characteristics and menstrual history of the participants.

Characteristics	Group		Test results
	Control	Ginger	
	Mean ± SD	Mean ± SD	
Age (year)	25.52 ± 3.13	24.76 ± 3.17	*P* = 0.33
Weight (kg)	59.61 ± 9.69	57.55 ± 7.4	*P* = 0.24
Height (cm)	160.9 ± 6.66	161.4 ± 5.72	*P* = 0.92
BMI (kg/m^2^)	23.33 ± 3.44	22.13 ± 2.95	*P* = 0.1
Age at menarche (year)	13.48 ± 1.25	13.21 ± 1.31	*P* = 0.39
Duration of cycle (day)	29 ± 1.42	28.7 ± 1.6	*P* = 0.46
Duration of menstruation (day)	5.3 ± 1.46	5.8 ± 1.36	*P* = 0.1

**Table 2:** Comparison of PMS scores in ginger and placebo groups before and after the intervention.

Symptoms	Group		Test results
	Control	Ginger	
	Mean ± SD	Mean ± SD	Unpaired test
Total severity of PMS			
Before intervention	106.7 ± 44.65	110.2 ± 30.77	*P* = 0.71
One month after intervention	105.7 ± 45.76	51.18 ± 32.76	*P* < 0.0001
Two months after intervention	107.2 ± 50.68	49.48 ± 33.12	*P* < 0.0001
Three months after intervention	106 ± 48.74	47.06 ± 33.41	*P* < 0.0001
Mood symptoms			
Before intervention	37.42 ± 17.37	38.97 ± 14.16	*P* = 0.69
One month after intervention	37.18 ± 16.13	16.39 ± 10.54	*P* < 0.0001
Two months after intervention	37.61 ± 18.58	14.61 ± 8.84	*P* < 0.0001
Three months after intervention	38.37 ± 20.31	13.45 ± 10.65	*P* < 0.0001
Physical symptoms			
Before intervention	42.64 ± 23.83	45.76 ± 19.76	*P* = 0.56
One month after intervention	43.48 ± 24.25	21.85 ± 22.54	*P* = 0.0004
Two months after intervention	43.42 ± 24.13	21.12 ± 20.31	*P* < 0.0001
Three months after intervention	42.06 ± 22.76	22.76 ± 19.62	*P* = 0.0005
Behavioral symptoms			
Before intervention	26.64 ± 16.20	25.42 ± 16.05	*P* = 0.76
One month after intervention	25.06 ± 17.86	12.94 ± 12.35	*P* = 0.002
Two months after intervention	26.15 ± 19.08	13.76 ± 13.79	*P* = 0.003
Three months after intervention	25.61 ± 18.47	10.85 ± 13.05	*P* = 0.0004
